# A Meta-Analysis of the Bacterial and Archaeal Diversity Observed in Wetland Soils

**DOI:** 10.1155/2014/437684

**Published:** 2014-05-28

**Authors:** Xiaofei Lv, Junbao Yu, Yuqin Fu, Bin Ma, Fanzhu Qu, Kai Ning, Huifeng Wu

**Affiliations:** ^1^Key Laboratory of Coastal Zone Environmental Processes and Ecological Remediation, Yantai Institute of Coastal Zone Research (YIC), Chinese Academy of Sciences (CAS), Shandong Provincial Key Laboratory of Coastal Zone Environmental Processes, YICCAS, Yantai 264003, China; ^2^University of Chinese Academy of Sciences, Beijing 100049, China

## Abstract

This study examined the bacterial and archaeal diversity from a worldwide range of wetlands soils and sediments using a meta-analysis approach. All available 16S rRNA gene sequences recovered from wetlands in public databases were retrieved. In November 2012, a total of 12677 bacterial and 1747 archaeal sequences were collected in GenBank. All the bacterial sequences were assigned into 6383 operational taxonomic units (OTUs 0.03), representing 31 known bacterial phyla, predominant with Proteobacteria (2791 OTUs), Bacteroidetes (868 OTUs), Acidobacteria (731 OTUs), Firmicutes (540 OTUs), and Actinobacteria (418 OTUs). The genus *Flavobacterium* (11.6% of bacterial sequences) was the dominate bacteria in wetlands, followed by Gp1, *Nitrosospira*, and *Nitrosomonas*. Archaeal sequences were assigned to 521 OTUs from phyla Euryarchaeota and Crenarchaeota. The dominating archaeal genera were *Fervidicoccus* and *Methanosaeta*. Rarefaction analysis indicated that approximately 40% of bacterial and 83% of archaeal diversity in wetland soils and sediments have been presented. Our results should be significant for well-understanding the microbial diversity involved in worldwide wetlands.

## 1. Introduction

Wetlands, which were estimated to be 45% of the total value of global natural ecosystems [1], are one of the most important terrestrial ecosystems and distribute in all regions throughout the world including Antarctica [2]. Microbiomes in wetlands play an important role in biogeochemical processes and microbial activities are crucial to the functions of wetland systems [3–8]. Moreover, microbial diversity is essential for exploiting potential of microbial resources from the wetland ecosystems [9–13]. It is crucial and necessary to understand the overall survival microorganisms in wetlands. Bacteria and archaea have been widely studied with respect to their biodiversity in natural and constructed wetlands [14–17]. Initial studies employed traditional culture-dependent methods and resulted in the discovery of plenty of new bacterial and archaeal taxa [18]. Employing kinds of molecular biology methods, increasing evidences have suggested that the structures of microbial communities are related to soil processes, such as cloning and sequencing of 16S rRNA genes, denaturing gradient gel electrophoresis (DGGE), terminal restriction fragment length polymorphism (T-RFLP), and quantitative PCR [4, 8, 19–23]. Cloning and sequencing of 16S rRNA genes have been widely used for their identification of potential known and unknown microbes [24]. Plenty of studies have examined the microbial diversity in wetlands using relatively large (>200 sequences) 16S rRNA clone libraries [4, 20, 25]. However, most studies to date have focused on individual wetland ecosystem [16, 26–28]. Many of the datasets published contain a small number of cloned sequences (generally >100), thus revealing only a small portion of the full diversity present in wetlands [10, 11, 29, 30]. The focus of some studies is limited to particular microbial group [31, 32]. In addition, there are many sequences recovered from wetlands with no additional information which were deposited into GenBank without being reported yet. High-throughput sequencing technologies, such as 454-pyrosequencing and ion torrent, were used to analyze the microbiomes in wetlands [30, 33–35]. These methods can produce huge datasets of short sequence reads. However, the length of these reads is too short to classify. Currently, there is no consensus on the size or nature of the microbial diversity generally found in wetlands. As a result, the understanding of the microbiomes in wetlands is fragmented and likely biased. This knowledge gap of microbiomes in wetlands will hamper the efficiency and stability of wetlands ecosystems. Few of the collective overviews of the microbial diversity in global wetlands are found up to date. The purposes of the study are to (1) perform a meta-analysis of all publicly available 16S rRNA gene sequences identified from various wetlands to provide a collective appraisal of the microbial diversity in wetland ecosystem, (2) make an effort to estimate the current coverage of the microbial diversity in wetlands, and (3) identify particular gaps in the knowledge and understanding of the microbial populations involved in wetlands.

## 2. Methods

### 2.1. Sequence Data Collection

Initial sequence sets were obtained from the GenBank (http://www.ncbi.nlm.nih.gov) and RDP (Release 10, http://rdp.cme.msu.edu) databases using the search terms (“wetland” OR “marsh” OR “fen”) AND “soil” AND “16S” on November 11, 2012. Non-16S rRNA sequences from GenBank were removed by checking the name of sequences. All 16S rRNA gene sequences from two databases were merged. Duplicate sequences identified based on accession numbers were removed. Mallared was used for checking sequences with vector nucleotides or chimera (http://www.softsea.com/review/Mallard.html). The 16S rRNA gene sequences of* Escherichia coli* (accession number: U00096) and* Methanothermobacter thermoautotrophicus* (accession number: AE000666) were selected as reference sequences for bacteria and archaea, respectively. In order to avoid uncertainties in comparing and classifying short sequences, sequences shorter than 250 bp were removed from the dataset which have few or no sequence overlap. The remaining sequences comprised the redacted composite dataset used in this work.

### 2.2. Phylogenetic Analysis

Sequences were aligned with Kalign [36] and classified into taxonomic ranks using the RDP Classifier with default settings [37]. Based on the output classifications from the RDP Classier, treemaps were constructed using the treemap packages in R. The dataset was divided into the following groups based on the classifications: Archaea, Bacteria, Proteobacteria, Actinobacteria, Firmicutes, Acidobacteria, Bacteroidetes, Chloroflexi, and the collected “minor phyla” of bacteria that comprised sequences not assigned to any of the aforementioned phyla. Distances matrices of aligned sequences were computed within ARB using Jukes-Cantor correction [38]. Individual distance matrices were analyzed using Mothur [39] to cluster OTUs, generated rarefaction curves, and estimated the expected maximum species richness complementary to the ACE and Chao1 richness. Unless otherwise stated, the genetic distance ≤0.03 was used to define species-level OTUs. The distance cut-off for other taxonomic ranks was set as follows: 0.05, genus; 0.10, family; 0.15, class/order; and 0.2, phylum. All the estimated asymptotes of the rarefaction curve were determined through R package monomol (https://github.com/binma/monomol) [40]. The coverage percentages were calculated as described by Nelson et al. [41].

### 2.3. Accession Numbers

The accession numbers for all sequences analyzed in this study were available from the corresponding author. The sequences were currently maintained in an in-house ARB database of 16S rRNA gene sequences for wetlands. A copy of this database and the sequence alignment were also available by request from the corresponding author.

## 3. Results and Discussion

This study was conducted as a meta-analysis ground on publicly available 16S rRNA gene sequences recovered from wetland soils worldwide. The sequences dataset collected from Genbank and RDP database was analyzed no matter their previously assigned taxonomic information or other analyses.

To address the long-term question of understanding microorganisms from wetland soil habitats, this study first aimed at characterizing prokaryotic communities inhabiting wetland soils. The prokaryotic microorganisms from wetland soil habitats drive the biogeochemical cycles of elements and may be a source of novel halophilic enzymes. Thus, we studied the diversity of prokaryotic microorganisms from wetland soils with meta-analysis approach.

### 3.1. Data Summary

**Figure 1:**
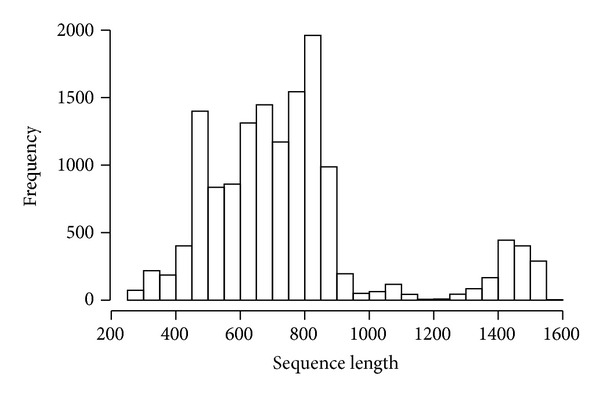
Distribution of the length of retrieved 16S rRNA sequences.

**Figure 2:**
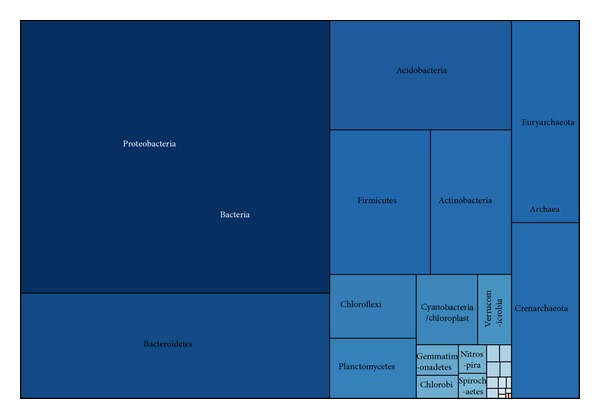
Treemap of observed prokaryotic taxons shown in their hierarchical order. Treemap showing taxonomic ranking of all taxa for all retrieved sequences. The size of each box is proportional to the number of sequences assigned to that taxon with respect to the entire dataset. The placement of boxes is arbitrary with respect to boxes within the same taxonomic rank and does not correspond to any form of phylogeny or relatedness.

Totally 14318 sequences longer than 250 bp were retrieved from GenBank and RDP databases. The sequences were mostly about 800 bp long, followed by approximately 600 bp (Figure 1). Interestingly, there is a small submit of sequence length between 1400 bp and 1600 bp. The 12583 bacterial sequences were assigned to 6383 OTUs, while the 1735 archaeal sequences were assigned to 521 OTUs (Table 1 and Figure 1). The most abundant bacterial and archaeal OTU contained 143 sequences and 113 sequences, respectively. Over 90% bacterial sequences were classified within five phyla, namely, Proteobacteria, Bacteroidetes, Acidobacteria, Firmicutes, and Actinobacteria (Figure 2). The remaining sequences were classified within 26 “minor” phyla, of which Chloroflexi, Planctomycetes, Cyanobacteria, and Verrucomicrobia were the only “minor” phyla with representation 1% of all bacterial sequences.

Of the archaeal sequences analyzed, all of them were classified within two phyla: Euryarchaeota and Crenarchaeota, representing 925 and 810 sequences, respectively.

### 3.2. Bacteria

#### 3.2.1. Proteobacteria

**Figure 3:**
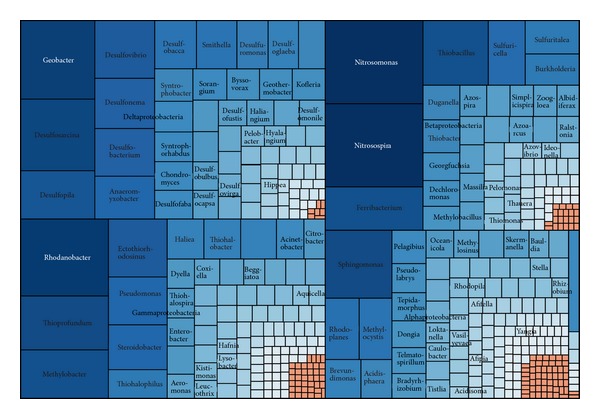
Treemap of observed Proteobacteria taxons shown in their hierarchical order.

The Proteobacteria was the largest and most diverse phylum in the present dataset. It comprised a total of 5637 sequences, approximately 44.8% of the bacterial sequences, assigned to 466 known genera. There are 2791 OTUs generated, with a Simpson diversity index of 0.0020. All six classes within the Proteobacteria were represented, but the Delta-, Gamma-, Beta-, and Alphaproteobacteria together represented over 99% of the proteobacterial sequences (Figure 3). The classes Epsilonproteobacteria and Zetaproteobacteria were extremely rare, represented by 43 and 1 sequences, respectively, indicating a low recovery rate in most of wetlands.

Classes in Proteobacteria showed various tendencies in different wetlands. The wide distribution of Gammaproteobacteria and Deltaproteobacteria in marine sediment has been documented, and most of them were involved in sulfur reduction under anaerobic conditions [4]. In comparison, a high abundance of Alphaproteobacteria and Betaproteobacteria appeared in freshwater sediment, and it is significantly correlated with pH and nutrients [34]. Some genera of Betaproteobacteria were confirmed to inhabit extremely alkaline wetland filled with historic steel slag [42]. The Epsilonproteobacteria is relatively abundant at oxic-anoxic interfaces such as intertidal wetland [43].

Deltaproteobacteria was the largest class in the phylum, with 1627 sequences (28.9% of the proteobacteria).* Geobacter* of family Geobacteraceae was the most abundant genus (9.8% of the Deltaproteobacteria) in Deltaproteobacteria. It was abundant in the rhizosphere and has been widely known as a kind of Fe (III)-reducing bacterium [44]. The followed abundant genera were* Deltaproteobacteria*,* Desulfosarcina*,* Desulfopila*,* Desulfovibrio*,* Desulfonema*, and* Desulfobacterium*, which represented greater than 1.0% of proteobacterial sequences. All of them were sulfate-reducing bacteria, and their distributions were influenced by salinity and plant nutrient [45]. They played important roles in the metabolism of nitrogen, phosphorus, sulfur, and some organic compounds in wetland systems [18, 46].* Anaeromyxobacter* was also the genus owning more than 1.0% proteobacterial sequences. As a kind of facultative bacteria, its unique respiratory reduction of nitrate and nitrite to ammonia was not linked to its ability to reduce nitrous oxide to nitrogen gas [47].

For the class Gammaproteobacteria, 1456 sequences were identified. It was the second largest class in Proteobacteria. Approximately 12.6% of gammaproteobacterial sequences (184 sequences) were assigned to the genus* Rhodanobacter* of family Xanthomonadaceae. This genus might be engaged in acidic denitrification in wetland soils [3]. The following abundant genera were* Thioprofundum* and* Methylobacter*, accounting for 8.9% (129 sequences) and 8.0% (108 sequences) of gammaproteobacterial sequences, respectively.* Thioprofundum* was recently considered as a mesophilic, facultatively anaerobic, sulfur-oxidizing bacterial strain [48].* Methylobacter* was reported as dominating in the Zoige wetland where the centers of methane emission were [24]. However, it was not affected by nitrogen leached from the catchment area in boreal littoral wetlands [9]. The other genera representing more than 1.0% proteobacteria sequences were* Ectothiorhodosinus*,* Pseudomonas*, and* Steroidobacter*.* Pseudomonas* was one of the widely studied PAH-degrading bacteria; it spread widely in contaminated wetlands environment [29] and was predominant microbial populations in the constructed wetland for nitrobenzene wastewater [32].

The 1420 betaproteobacterial sequences were identified in Proteobacteria. The genus* Nitrosomonas* was the predominant genera with 222 assigned sequences, while the genus* Nitrosospira* was the second abundant genus with 217 sequences. They were also the first and second most abundant proteobacterial genera, and both of them belonged to the family Nitrosomonadaceae which were well known as the main ammonia-oxidizing microorganisms contributing to N_2_O production in wetlands and sediments [31, 49, 50]. The genera* Ferribacterium*,* Thiobacillus*, and* Sulfuricella* owned more than 1.0% of proteobacterial sequences.

The fourth largest proteobacterial class was Alphaproteobacteria, with 1090 sequences (over 19.3%). The dominating genus* Sphingomonas* in class Alphaproteobacteria was widely distributed in wetland and sediments, due to its ability to survive in low concentrations of nutrients, as well as to metabolize a wide variety of carbon sources [7, 51]. Except for* Sphingomonas* which contains over 2.0% of the proteobacterial sequences (122 sequences), other genera of Alphaproteobacteria represented less than 1.0% proteobacteria sequences.

#### 3.2.2. Bacteroidetes

**Figure 4:**
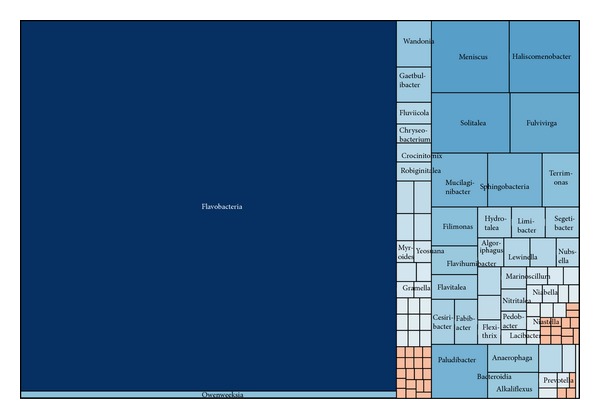
Treemap of observed Bacteroidetes taxons shown in their hierarchical order.

Bacteroidetes was the second abundant phylum in the present dataset, including 2244 sequences (nearly 17.8% of all bacterial sequences), which were assigned to 109 known genera, with 868 OTUs and a Simpson diversity index of 0.0007 (Figure 4). A plenty of Bacteroidetes strains isolated from wetland soils and sediments were reported to be anaerobic and saprophytic representative bacteria [52, 53]. Highlighting the unevenness of the phylum, over 70% of all the Bacteroidetes sequences (12.8% of all bacterial sequences) were assigned to class Flavobacteria. As a common heterotrophic obligate aerobe, Flavobacteria was the second largest class in the dataset. It is widespread in various wetlands, even in swine wastewater lagoon and constructed wetlands [54, 55]. The class Sphingobacteria was represented by only 491 sequences, while the class Bacteroidia was represented by only 75 sequences. “Undefined Bacteroidetes” comprised 65 sequences.

The most frequently observed genus in Flavobacteria was* Flavobacterium* (1459 sequences), which was also the most abundant bacterial genus in this dataset. A number of species of* Flavobacterium* have been isolated from rhizosphere of wetland [52, 53].

#### 3.2.3. Acidobacteria

**Figure 5:**
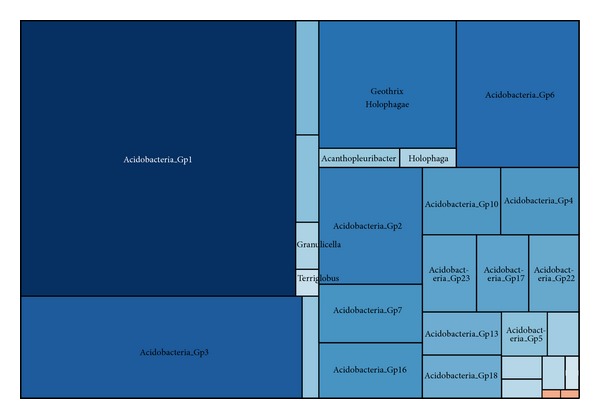
Treemap of observed Acidobacteria taxons shown in their hierarchical order.

Acidobacteria was the third largest phylum in our dataset, including 1345 sequences assigned to 29 genera. Acidobacteria is a new phylum, whose members are physiologically diverse and ubiquitous in soils, but are underrepresented in culture at present. There were 731 OTUs identified, with a Simpson diversity index of 0.0031 (Figure 5). Just over 90% of all the acidobacterial sequences (9.7% of all bacterial sequences) were assigned to 21 unclassified groups, only 130 sequences represented to class Holophagae. In total, nearly 40% of the Acidobacteria sequences were able to be classified to Gp1, which was the second largest class of bacteria. The following classes were group Gp3 and then group Gp6, with 196 and 116 sequences, respectively. As the reports, Acidobacteria group was more abundant in natural wetlands than in created wetlands [10, 34], especially in freshwater sediment [34]. Acidobacteria has been reported as the largest division in the active layer and the associated permafrost of a moderately acidic wetland in Canada [11]. Future studies are needed to examine the interrelations of environmental parameters with Acidobacteria and individual populations within subgroups [56].

#### 3.2.4. Firmicutes

**Figure 6:**
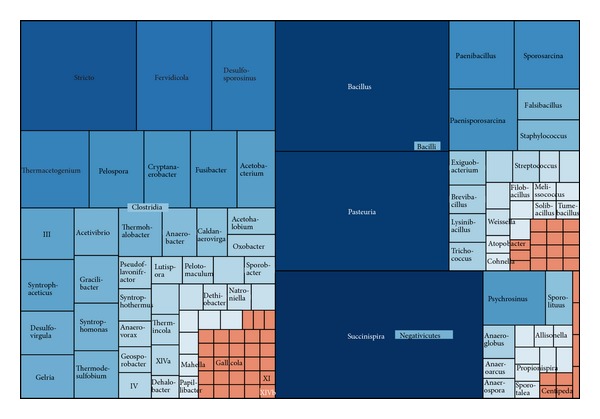
Treemap of observed Firmicutes taxons shown in their hierarchical order.

The fourth largest phylum was the Firmicutes, assigned into 973 sequences and 540 OTUs with a Simpson diversity index of 0.0041 (Figure 6). As saprophytic microbes, some members of Firmicutes are known to produce endospores under stressful environmental conditions such as in intertidal sediment [34], extremely alkaline (pH > 12) constructed wetland [42].

In total, about 45% of the Firmicutes sequences were classified to the class Clostridia, and nearly 36% were classified into the class Bacilli. The Clostridia (sulfite-reducing bacteria) is an anaerobic and highly polyphyletic bacterium, while Bacilli can be obligate aerobes or facultative anaerobes. There was a long record of evidence to suggest that both of them were the abundant taxa in sewage sludge [57]. Some species of them exhibit great ability to degrade hydrocarbons in crude oil contaminated wetland ultisol [6]. Within the class Bacilli, two primary genera were* Bacillus* and* Pasteuria*, representing 107 and 98 sequences, respectively. While in Clostridia, genus* Stricto* was the most abundant genus, with 56 sequences.

The class Negativicutes represented 178 sequences. The genus* Succinispira* represented over 70% of sequences in Negativicutes. The genus* Succinispira*, the most abundant genus in Firmicutes, was capable of decarboxylating succinate in anaerobic conditions. The class Erysipelotrichia represented only three sequences.

#### 3.2.5. Actinobacteria

**Figure 7:**
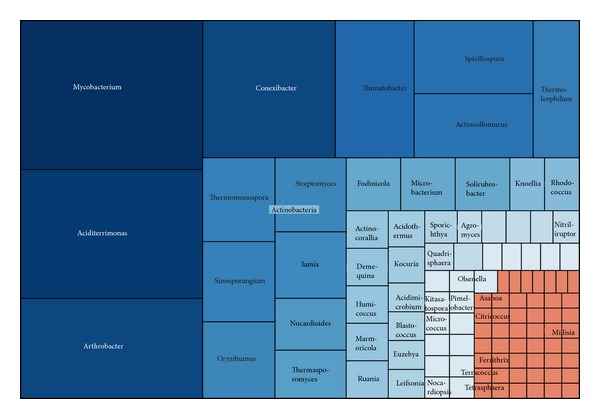
Treemap of observed Actinobacteria taxons shown in their hierarchical order.

As the fifth abundant phylum, Actinobacteria represented 783 sequences, clustered into 418 OTUs, with a Simpson diversity index of 0.0054. All of Acidobacteria sequences were classified to the class Actinobacteria and over 66% of them belonged to order Actinomycetales (Figure 7). Actinobacteria can be terrestrial or aquatic, playing an important role in the decomposition of organic materials. Although understood primarily as soil bacteria, they might be more abundant in freshwaters [10, 57].

*Mycobacterium* (103 sequences) was the most frequently observed genus in Actinobacteria. It has been widely detected from contaminated soil or sediments [51]. Some species of* Mycobacterium* were the dominant PAH-degraders and played an important role in degrading PAHs in contaminated mangrove sediments [7]. The following abundant genera were* Aciditerrimonas*,* Conexibacter*,* Arthrobacter*, and* Ilumatobacter*. The rest of genera were less than 5% of actinobacterial sequences.

#### 3.2.6. Minor Phyla

In addition to the five phyla described above, 26 minor phyla with 1601 sequences were also observed based on the dataset. Of these minor phyla, only the phyla Chloroflexi (2.96%), Planctomycetes (2.77%), Cyanobacteria (2.28%), and Verrucomicrobia (1.28%) represented more than 1% of all the bacterial sequences and accounted for over 73% of all minor phyla sequences (Figure 1).

Some known genera were represented in these “minor phyla.” The most abundant of the minor phyla, Chloroflexi, comprised 372 sequences. Members of the Chloroflexi are generally found in intertidal sediment and moderately acidic wetland [11, 13, 34, 58]. Planctomycetes was the second most abundant of the minor phyla, to which 349 sequences were assigned. A number of genera of the Planctomycetes, which were once thought to occur primarily in aquatic environments, have been discovered in wetlands [12, 29]. As the third most abundant minor phyla, Cyanobacteria occupy a broad range of habitats across all latitudes. They are widespread in freshwater, marine, and even in the most extreme niches such as hot springs and hypersaline bays [12, 59, 60]. Evidence suggests that Verrucomicrobia are abundant within the environment and important. The species of Verrucomicrobia have been identified and isolated from fresh water and soil environments [61].

### 3.3. Archaea

#### 3.3.1. Euryarchaeota

**Figure 8:**
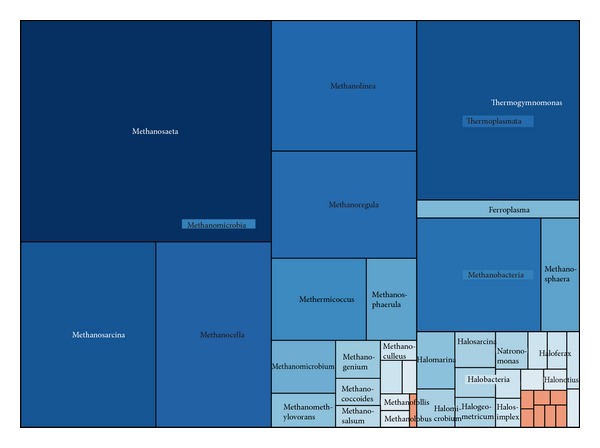
Treemap of observed Euryarchaeota taxons shown in their hierarchical order.

Euryarchaeota comprised 925 sequences, approximately 53.3% archaeal sequences. They were clustered into 418 OTUs with a Simpson diversity index of 0.0054 (Figure 8). The majority (70.9%) of Euryarchaeota sequences were assigned to the methanogenic class Methanomicrobia (656 sequences). The class Thermoplasmata comprised 132 sequences, while the class Methanobacteria comprised 75 sequences. Only 59 sequences were classified into class Halobacteria. Classes Archaeoglobi and Methanopyri represented only 2 and 1 sequences, respectively.

Methanomicrobia contributes a large proportion of methane emission in wetlands, no matter in cold area or in subtropical places [19, 62]. As seen in Figure 6, the most predominate Methanomicrobia genus (223 sequences) was* Methanosaeta* (formerly* Methanothrix*), which was also the second most abundant archaeal genus. It was reported precisely as the dominant acetoclastic methanogen in the high arctic wetlands [63]. The methanogens genera* Methanosarcina*,* Methanocella*,* Methanolinea*, and* Methanoregula* each represented nearly 10% of Euryarchaeota sequences. The other 12 genera were only represented by a small number of sequences in the dataset.

The largest genus in class Thermoplasmata was* Thermogymnomonas* (120 sequences), which was detected widely even at low pH wetlands. It was known as a kind of iron-oxidizing microorganisms [64]. The rest 12 sequence of Euryarchaeota were assigned to genus* Ferroplasma*, an anaerobic and acidophilic archaea, which coupled to the reduction of ferric iron [5]. Of the class Methanobacteria, there were two genera,* Methanobacterium* and* Methanosphaera*, with 57 and 18 sequences, respectively. Both of these genera were detected from water and sediments of a high-altitude athalassohaline wetland [25]. As a facultative anaerobic archaea, Halobacteria was common in most environments where large amounts of salt, moisture, and organic material are available [25].

#### 3.3.2. Crenarchaeota

Crenarchaeota owned less abundant sequences than Euryarchaeota in the dataset, with 810 sequences. Crenarchaeota diversity was lower, with only 197 OTUs generated and a Simpson diversity index of 0.0443. It suggested that Crenarchaeota was more related to aerobic metabolisms in the water and surface sediment [65].

**Figure 9:**
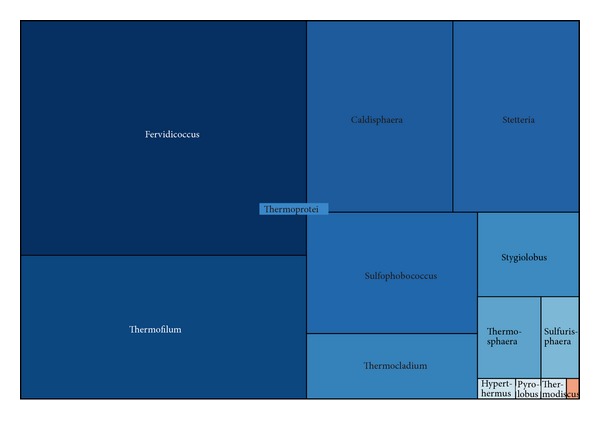
Treemap of observed Crenarchaeota taxons shown in their hierarchical order.

All of the Crenarchaeota sequences were assigned to the class Thermoprotei (Figure 9). As the reports, class Thermoprotei dominated in archaeal phyla in Pacific influenced sediments, while Methanomicrobia inhabited in methane-containing Atlantic influenced sediments [58]. Within the class, 258 sequences were classified to the genera* Fervidicoccus*.* Fervidicoccus* was the most abundant genera in archaeal and has been cultivated and characterized widely. The following abundant genera in this phylum were* Thermofilum* (19.5%),* Caldisphaera* (13.3%), and* Stetteria* (11.5%). The other genera sequences were less than 10%.

### 3.4. Diversity Estimates

For all of the bacterial groups, the ACE value of richness was the greatest, while the majority of corresponding rarefaction estimates were the lowest (Table 1). Similar with rarefaction estimates, the ACE and Chao1 estimate the maximum species richness for an OUT definition. However, the richness estimates derived from the rarefaction curves differed less from the Chao1 estimate, comparing with those from ACE estimates. The richness estimates derived from ACE differed greatly (72~120%) for Bacteria, Proteobacteria, Acidobacteria, Firmicutes, and Actinobacteria, while the corresponding estimates for the Bacteroidetes, Archaea, Euryarchaeota, and Crenarchaeota were less than 70% different.

**Figure 10:**
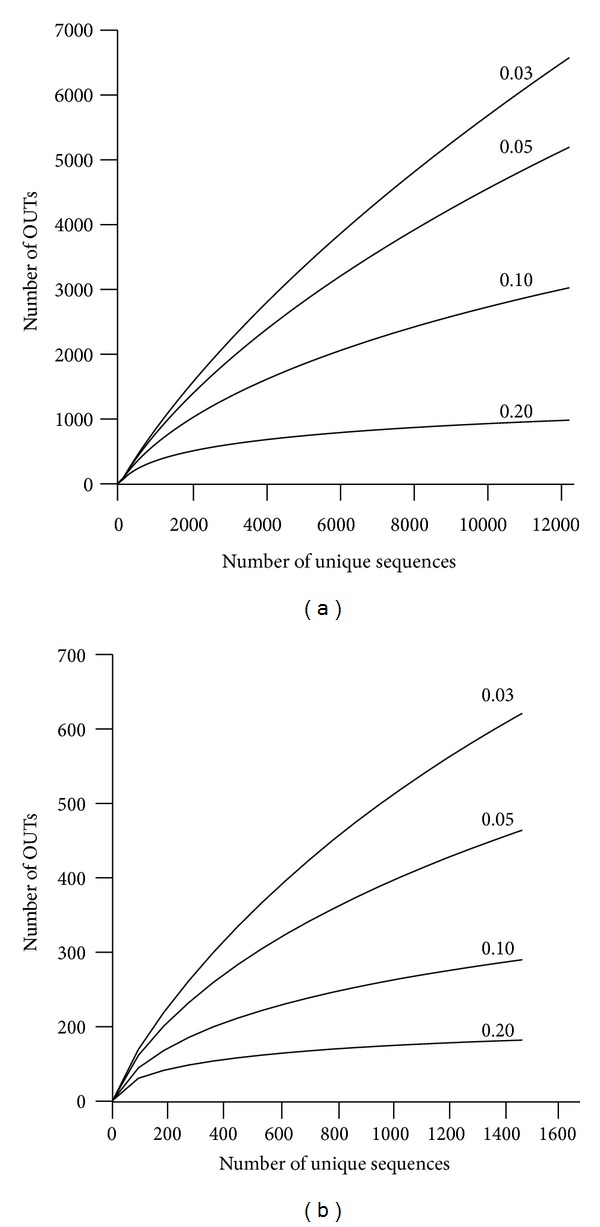
Rarefaction curve for the Archaea (a) and Bacteria (b) with different dissimilarity cut-off.

The present results showed that the coverage of microbial diversity in wetlands was remaining rather low. Rarefaction analysis of Bacteria showed that only sampling at the phylum (0.20 phylogenetic distance) level has begun to reach a horizontal plateau. The other sampling at the taxonomic ranks was still projecting upward (Figure 10 and Table 2). At the species (0.03 phylogenetic distance) level, only 41% of the expected diversity has been revealed. The estimates of current coverage suggest that of Bacteria was less than that of Actinobacteria, Bacteroidetes, and Firmicutes, greater than that of Acidobacteria. Coverage rate of Proteobacteria was similar to that of the Bacteria. For the archaea, the coverage of diversity was greater than bacteria, but still low compared to estimated richness. There was about 59% of the expected diversity revealed at the species level. The estimates of current coverage of Euryarchaeota and Crenarchaeota were much greater than that of Archaea. As the results of rarefaction analysis and diversity statistics, it was obvious that the known bacterial and archaeal diversity in wetlands were incomplete below the phylum level. Nevertheless, the global microbial diversity in wetlands revealed in this study had ability to serve as a framework for future studies of alpha and beta diversity. More specifically, the collected sequence dataset could give a hand on detecting and quantifying specific groups of either bacteria or archaea at the nucleotide level. Additionally, these studies will greatly advance the ecology of individual microbia collected in the dataset.

Sufficient coverage and depth were provided to explore an individual sample or compare multiple samples through multiplexing, with the developing of second generation sequencing technologies. Moreover, new sequences dataset could be added to the composite datasets analyzed in this study to increase our knowledge on the diversity of this ecosystem. The knowledge on the diversity may shine light on the understanding of the microbiomes of wetlands and define the significance of individual microbia. It is also suited for continuous following of the succession variation of the diversity of wetlands. However, the beta diversity was hardly determined because most of studies could not contain large sequence datasets and detailed information with same methodologies and sequence submission criteria. A “core group” was defined after analyzing seven municipal sludge digesters [66]. Although distinct microbiomes are possibly being selected under a unique environment, only a small number of “core OTUs” can be found among the large numbers of OTUs identified. Systematic studies examining multiple wetlands designs with great depth of coverage should help further define the “core microbiomes” in wetlands.

Now that analysis of 16S rRNA gene sequences can provide insight into the functional diversity of wetlands, the metabolic functions of organisms are getting more concerned. For a good comprehension of the metabolic capacities of these organisms, metagenomic studies techniques such as SIP and MAR-FISH should be used more frequently. Cultivation-based studies are also needed to define the functions of uncharacterized species of bacteria and archaea in wetlands.

## 4. Conclusions

The present dataset generated from GenBank and RDP databases was largely dominated by Proteobacteria. Approximately 40% of sequences and OTUs belonged to Proteobacteria. Our results showed that (1) nearly 56% of the archaeal and 45% of the bacterial species-level diversity in wetlands have been witnessed; (2) sequences from the bacterial phyla Proteobacteria, Bacteroidetes, Chloroflexi, Firmicutes, Actinobacteria, and archaeal class were well represented by the available sequences and the corresponding microorganisms were probably important participants in the wetland environments; (3) the global diversity contains numerous groups for which there was no close cultured representative, especially the majority of sequences assigned to the phyla Chloroflexi and Bacteroidetes. Therefore future studies should utilize multiple approaches to characterize the microbial diversity and its function in wetlands.

## Figures and Tables

**Table 1:** Diversity statistics for Archaea, Bacteria, and “Major” phylum groups. Coverage = #OTUs/rarefaction estimate; OTU and abundance were calculated using a 0.03 dissimilarity cut-off.

Group	Total sequences	Unclassified to phylum	Number of OTUs	ACE	Chao1	Rarefaction estimation	Current coverage (%)
Bacteria	12583	none	6383	30581	17176	15768	41
Pro	5763		2791	12472	7245	6811	40
Act	783		418	2280	1088	1033	46
Aci	1345		731	2972	1693	1602	28
Fir	973		540	3595	1856	1915	54
Bact	2244		868	2700	1887	1601	59
Archaea	1735	none	521	1131	884	883	83
Eur	925		418	681	505	504	62
Cre	810		197	442	311	320	41

**Table 2:** Estimates of current taxonomic coverage for Archaea and Bacteria.

Distance	Number of Current OTUs	Rarefaction estimation	Coverage^a^ (%)
Archaea			
0.03	521	883	59
0.05	364	587	62
0.10	190	278	68
0.20	82	91	90
Bacteria			
0.03	6383	15768	40
0.05	5042	9854	51
0.10	2937	4617	63
0.20	954	1118	85

^a^Coverage = number of OTUs/rarefaction estimate.
